# Supplementary figures and images for: The Walking Behaviour of Pedestrian Social Groups and Its Impact on Crowd Dynamics
Source: PLoS One. 2010 Apr 7;5(4):e10047. doi: 10.1371/journal.pone.0010047 (PMC2850937; doi:10.1371/journal.pone.0010047)

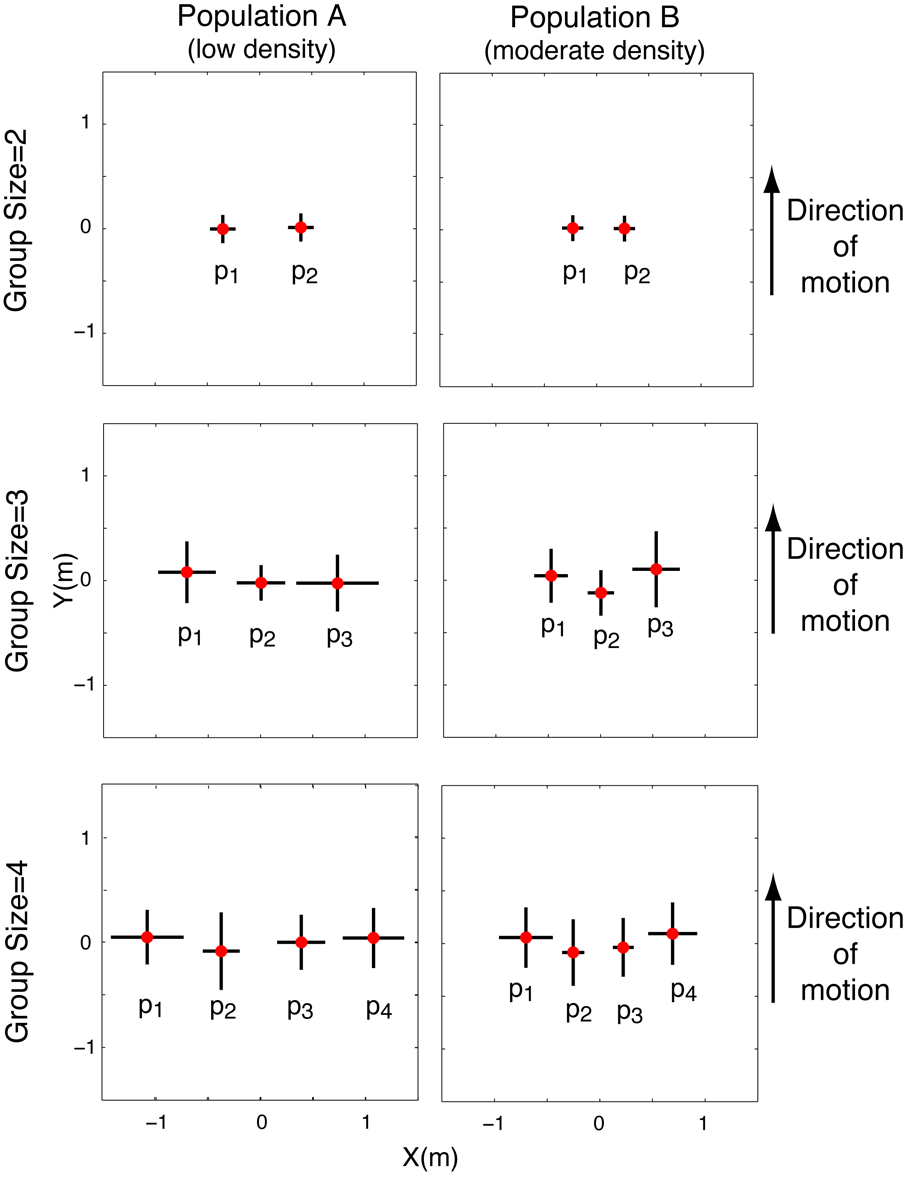

Supplement: Figure S1 — Observed patterns of spatial organization. The group's centre of mass is located at the origin and the red points indicate the average positions of group members. The black bars indicate the standard deviation of the average positions along the x and y axes. (3.23 MB TIF) [file pone.0010047.s001.tif]
