# Supplementary material for: The Walking Behaviour of Pedestrian Social Groups and Its Impact on Crowd Dynamics
Source: PLoS One. 2010 Apr 7;5(4):e10047. doi: 10.1371/journal.pone.0010047 (PMC2850937; doi:10.1371/journal.pone.0010047)
Supplement: Table S1 — Comparison between observed and simulated patterns. The table shows the results of a series of t-tests comparing the observed and predicted distributions of angle αij for group size two, three and four in population A and B. (0.05 MB DOC) [file pone.0010047.s002.doc]

|  |  | **Population A** | **Population B** |
| --- | --- | --- | --- |
| **Size=2** |  | p>0.5 | p>0.5 |
| **Size=3** |  | p=0.10 | p=0.33 |
|  |  | p=0.10 | p=0.28 |
| **Size=4** |  | p>0.5 | p=0.07 |
|  |  | p>0.5 | p=0.15 |
|  |  | p=0.22 | p=0.07 |

**Table S1:** Comparison between observed and simulated patterns. The table shows the results of a series of t-tests comparing the observed and predicted distributions of angle $\alpha_{\text{ij}}$ for group size two, three and four in population A and B.
